# New tricks of an old enemy: isolates of *F* *usarium graminearum* produce a type A trichothecene mycotoxin

**DOI:** 10.1111/1462-2920.12718

**Published:** 2015-01-30

**Authors:** Elisabeth Varga, Gerlinde Wiesenberger, Christian Hametner, Todd J. Ward, Yanhong Dong, Denise Schöfbeck, Susan McCormick, Karen Broz, Romana Stückler, Rainer Schuhmacher, Rudolf Krska, H. Corby Kistler, Franz Berthiller, Gerhard Adam

**Affiliations:** ^1^ Christian Doppler Laboratory for Mycotoxin MetabolismCenter for Analytical ChemistryDepartment for Agrobiotechnology (IFA‐Tulln)University of Natural Resources and Life Sciences, Vienna (BOKU)Konrad Lorenz Str. 203430TullnAustria; ^2^ Department of Applied Genetics and Cell BiologyUniversity of Natural Resources and Life Sciences, Vienna (BOKU)Konrad Lorenz Str. 243430TullnAustria; ^3^ Institute of Applied Synthetic ChemistryVienna University of TechnologyGetreidemarkt 9/163‐OC1060ViennaAustria; ^4^ Bacterial Foodborne Pathogens and Mycology Research UnitNational Center for Agricultural Utilization ResearchUnited States Department of Agriculture1815 N. University StreetPeoriaIL61604USA; ^5^ Department of Plant PathologyUniversity of Minnesota1991 Upper Buford CircleSt PaulMN55108USA; ^6^ Agriculture Research ServiceUnited States Department of Agriculture1551 Lindig AvenueSt PaulMN55108USA

## Abstract

The ubiquitous filamentous fungus *F* *usarium graminearum* causes the important disease Fusarium head blight on various species of cereals, leading to contamination of grains with mycotoxins. In a survey of *F* *. graminearum* (*sensu stricto*) on wheat in North America several novel strains were isolated, which produced none of the known trichothecene mycotoxins despite causing normal disease symptoms. In rice cultures, a new trichothecene mycotoxin (named NX‐2) was characterized by liquid chromatography‐tandem mass spectrometry. Nuclear magnetic resonance measurements identified NX‐2 as 3α‐acetoxy‐7α,15‐dihydroxy‐12,13‐epoxytrichothec‐9‐ene. Compared with the well‐known 3‐acetyl‐deoxynivalenol (3‐ADON), it lacks the keto group at C‐8 and hence is a type A trichothecene. Wheat ears inoculated with the isolated strains revealed a 10‐fold higher contamination with its deacetylated form, named NX‐3, (up to 540 mg kg^−1^) compared with NX‐2. The toxicities of the novel mycotoxins were evaluated utilizing two *in vitro* translation assays and the alga *C* *hlamydomonas reinhardtii*. NX‐3 inhibits protein biosynthesis to almost the same extent as the prominent mycotoxin deoxynivalenol, while NX‐2 is far less toxic, similar to 3‐ADON. Genetic analysis revealed a different *TRI* *1* allele in the N‐isolates, which was verified to be responsible for the difference in hydroxylation at C‐8.

## Introduction

**Figure 1:**
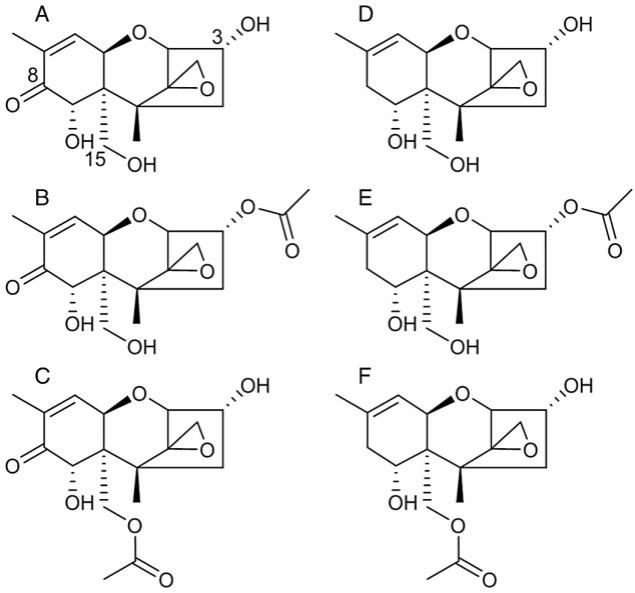
Structures of (A) deoxynivalenol, (B) 3‐acetyl‐deoxynivalenol, (C) 15‐acetyl‐deoxynivalenol as well as of the novel metabolites (D) NX‐3, (E) NX‐2 and (F) NX‐4.

The growth of fungi on agricultural goods is a worldwide problem, limiting available food and feed supplies. The Food and Agriculture Organization of the United Nations (FAO) estimates that about a quarter of the world's food crops are significantly contaminated with mycotoxins (Smith *et al*., 1994). Mycotoxins are secondary fungal metabolites of low molecular weight, which are toxic to humans and animals (Bennett and Klich, 2003; Marin *et al*., 2013). Deleterious health effects caused by different mycotoxins include nephropathy, infertility, cancer or death. One of the main classes of mycotoxins are trichothecenes (McCormick *et al*., 2011). This family encompasses around 200 different toxins, which inhibit eukaryotic protein synthesis (Cundliffe and Davies, 1977). They are therefore also potent phytotoxins and act as virulence factors of pathogenic fungi on sensitive host plants, e.g. of *Fusarium graminearum* in wheat (Bai *et al*., 2002; Jansen *et al*., 2005). Although all trichothecenes share a tricyclic 12,13‐epoxytrichothec‐9‐ene structure (Fig. 1), they can be further classified according to their substituents on C‐8. Type A trichothecenes are characterized by a hydroxyl group, an ester or no substituent at all at C‐8, whereas type B trichothecenes carry a keto group at this position (McCormick *et al*., 2011).

Although several fungi can form trichothecenes, the by far most relevant genus regarding pathogenicity on cereals is *Fusarium*, with *F. graminearum* being the dominant species causing Fusarium head blight disease (scab) (reviewed in Goswami and Kistler, 2004; Kazan *et al*., 2012). This disease occurs worldwide, but its dramatic reappearance in North America during the 1990s had a particularly severe impact on US farming communities (McMullen *et al*., 1997). Because of reduced yields and market prices for mycotoxin contaminated grain, growers had to give up planting wheat and brewing barley in severely affected areas (McMullen *et al*., 2012).

The most frequently occurring mycotoxin due to *F. graminearum* infection is the type B trichothecene deoxynivalenol (DON, Fig. 1A), along with its biosynthetic precursors 3‐acetyl‐DON (3‐ADON, Fig. 1B) and 15‐acetyl‐DON (15‐ADON, Fig. 1C). The currently known mechanisms of action and the toxicological relevance of DON are reviewed (Pestka, 2010). It causes immunosuppressive, emetic and anorexic effects after ingestion. Occurrence data for DON, collected by 21 European countries between 2007 and 2012, and population exposure was recently summarized by the European Food Safety Authority (2013). Several dozen countries, including Canada, China, the European Union, India, Japan and Russia, set maximum limits for DON in different foodstuffs (FAO, 2006). In the European Union, the maximum level (European Commission Regulation, 2006) for DON in unprocessed cereals is set at 1.25 mg kg^−1^, while cereals intended for direct human consumption may contain up to 0.75 mg kg^−1^. The US Food and Drug Administration set advisory levels for DON in finished wheat products for human consumption (1 mg kg^−1^) as well as for grains and grain by‐products used for animal feed (US Food and Drug Administration, 2010).

*Fusarium* isolates can be classified according to their chemotype (Moss and Thrane, 2004) based on which toxin accumulates in axenic cultures. Different *TRI8* alleles result either in the deacetylation of the biosynthetic precursor 3,15‐diacetyl‐DON at C‐15 to yield 3‐ADON or at C‐3 to yield 15‐ADON (Alexander *et al*., 2011). While the 15‐ADON chemotype of *F. graminearum* used to be more prevalent in North America, the frequency of the 3‐ADON chemotype in western Canada increased 14‐fold between 1998 and 2004 (Ward *et al*., 2008). The biosynthetic enzymes required for trichothecene production are encoded by *TRI* genes at three different loci (*TRI101* locus, *TRI1* and *TRI16* locus and the 12 gene core *TRI* cluster) (McCormick *et al*., 2011). *TRI13* is responsible for the introduction of a hydroxyl group at C‐4 of the trichothecene skeleton, e.g. leading to the type B trichothecene nivalenol (NIV), which is more prevalent in Asia.

Strains of a newly identified population of *F. graminearum* (*sensu stricto*), collected from wheat in Minnesota, were genotyped to be 3‐ADON producers, but chemical analysis showed that they produced neither DON nor NIV nor acetylated derivatives thereof (Gale *et al*., 2010). Rice cultures of the isolated strains were screened with a multi‐mycotoxin method (Vishwanath *et al*., 2009), confirming the absence of all trichothecenes included in the method. These ‘no trichothecene’ producers were previously investigated as potential biocontrol strains, as co‐inoculation of wheat with a DON producer reduced the DON content (Yuen *et al*., 2010).

The aim of our work was to investigate whether the isolated *F. graminearum* strains can produce novel trichothecene toxins because clear disease symptoms could be seen on wheat. Here we report on the identification and characterization of two novel mycotoxins (NX‐3 Fig. 1D, NX‐2 Fig. 1E) produced by these strains. The toxicity of these compounds renders our work potentially important for food safety.

## Results

### Identification of new trichothecenes

Gas chromatography‐mass spectrometry (GC‐MS) headspace (HS) analysis of isolates previously identified as ‘Northland’ (Gale *et al*., 2010) or ‘No trichothecene’ (N) showed that they produce the volatile trichothecene precursor trichodiene, suggesting that unknown trichothecenes might be produced by the N‐strains.

**Figure 2:**
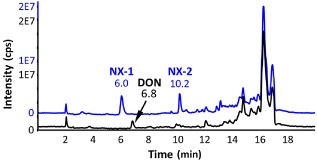
LC‐MS full scan comparison of *F* *. graminearum* PH‐1 (black line) and a *F* *. graminearum* N‐isolate (blue line).

Full‐scan liquid chromatographic‐mass spectrometric (LC‐MS) measurements of the extracts from rice cultures of an N‐isolate and a control strain, PH‐1, identified two candidate compounds with significant intensity (Fig. 2). The first eluting peak of *m/z* 342 was slightly more polar than DON and was termed NX‐1. The second one (*m/z* 324) was about as polar as 3‐ADON and was named NX‐2. The substances were purified from 24‐day‐old rice cultures of isolate 06‐156 via normal phase and subsequent reversed phase chromatography. Further liquid chromatographic high‐resolution mass spectrometric (LC‐HRMS) measurements revealed a sum formula of C_17_H_24_O_6_ for NX‐2 ([M + H]^+^ calculated. for C_17_H_24_O_6_, 325.1646; found, 325.1647; [M+NH_4_]^+^ calculated. 342.1911; found, 342.1915; [M+Na]^+^ calculated. 347.1465; found, 347.1467) The obtained HRMS/MS spectra indicated the presence of an acetyl group and at least two hydroxyl groups in NX‐2 (Fig. S1A). NX‐1 was heavier than NX‐2 by two protons and one oxygen atom.

Structure elucidation and signal assignment was carried out based on 1D nuclear magnetic resonance (NMR) (^1^H, ^13^C‐CPD or ^13^C‐APT) and 2D NMR (^1^H^1^H correlation spectroscopy, ^1^H^13^C heteronuclear single quantum correlation and ^1^H^13^C hetero‐nuclear multiple bond correlation). 1D nuclear Overhauser effect (NOE) difference or 2D‐nuclear Overhauser effect spectroscopy (NOESY) spectra were recorded to elucidate stereochemistry. The chemical structure was elucidated as 3α‐acetoxy‐7α,15‐dihydroxy‐12,13‐epoxytrichothec‐9‐en (Fig. 1E, 7‐hydroxy‐15‐decalonectrin) and hence showed the characteristic backbone structure of trichothecenes. NMR data are summarized in Table S1 for ^1^H and in Table S2 for ^13^C. Compared with 3‐ADON, NX‐2 lacks the keto‐group at C‐8 characteristic for type B trichothecenes and hence is classified as a type A trichothecene. NX‐1 is a derivative of NX‐2 (addition of water) very similar to the previously described DAS‐M1 (Shams *et al*., 2011) and therefore assumed to be a preparation artefact.

3‐ADON and 15‐ADON can be rapidly hydrolysed to DON *in planta*, and DON is the major form found in inoculated wheat. Suspecting a similar reaction for NX‐2, its deacetylated product (named NX‐3, Fig. 1D) was produced by alkaline hydrolysis and subsequent purification via preparative high‐performance liquid chromatography (HPLC). The structure of NX‐3 was also confirmed by HRMS ([M+H]^+^ calculated. for C_15_H_22_O_6_, 283.1540; found, 283.1540; [M+Na]^+^ calculated. 305.1359; found, 305.1365) and NMR (see Table S1 for ^1^H‐NMR and in Table S2 for ^13^C‐NMR).

### Formation of the new trichothecenes in planta

The highly sensitive wheat cultivar ‘Apogee’ (Mackintosh *et al*., 2006) was grown in a growth chamber (19 h light per day, 20°C, rel. humidity 55%), and the ears were spray inoculated with suspensions of *F. graminearum* PH‐1 (as control) and different N‐isolates (02‐264, 06‐146, 06‐156 and 06‐171). Samples were harvested after 11, 16 and 22 days. Liquid chromatographic‐tandem mass spectrometric (LC‐MS/MS) analyses revealed that DON was present in the control sample in high amounts (> 30 mg kg^−1^), and none of the novel trichothecenes were detected. In the wheat ears treated with the N‐isolates, approximately 10 times more NX‐3 than NX‐2 was detected (Table 1) and no DON was found. Similar results were obtained with these and further isolates on wheat cv. ‘Norm’ in a glasshouse experiment. Again the major compound was NX‐3, with NX‐2 occurring around 10 times less. No NX‐1 was detected in any of the analysed wheat samples. Because GC‐MS instruments are often used for the determination of trichothecenes in cereals, wheat heads inoculated with the *F. graminearum* isolates were also analysed using this technique. A dominant peak at 6.08 min was observed in GC‐MS, matching the retention time of the trimethylsilyl (TMS) derivative of NX‐3. The mass spectrum TMS‐NX‐3 is shown in Fig. S2. The compound produces a weak molecular ion at *m/z* 498 (M^+^) (Fig. S2, insert), which is confirmed by an intense [M+H]^+^ ion at *m/z* 499.2720 by positive chemical ionization (PCI) high‐resolution GC‐MS. The molecular formula of TMS‐NX‐3 is verified as C_24_H_46_O_5_Si_3_. The existence of three hydroxyl groups is confirmed by the formation of fragment ions at 408.2152 *m/z* (elimination of one TMSOH), 318.1649 *m/z* (elimination of two TMSOH) and 305.1568 *m/z* (loss of one TMSOH and one [CH_2_ = OSi(CH_3_)_3_] group). The mass spectrum of TMS‐NX‐3 also shows intense peaks at *m/z* 103, 147 and 181, which are common features for DON and its trichothecene analogues (Rodrigues‐Fo *et al*., 2002). While a second peak at a retention time of 6.66 min was observed for TMS‐NX‐2, no NX‐1 was found in the wheat samples.

**Table 1:** Concentration of NX‐2 and NX‐3 in wheat ears inoculated with different N‐isolates

	02‐264			06‐146			06‐156			06‐171		
	11d	16d	20d	11d	16d	20d	11d	16d	20d	11d	16d	20d
NX‐2 (mg kg^‒1^)	55	25	46	30	21	62	45	38	42	30	34	34
NX‐3 (mg kg^‒1^)	480	190	260	220	260	540	330	350	460	270	380	370

### *In vitro* translation inhibition and phytotoxicity assays

**Figure 3:**
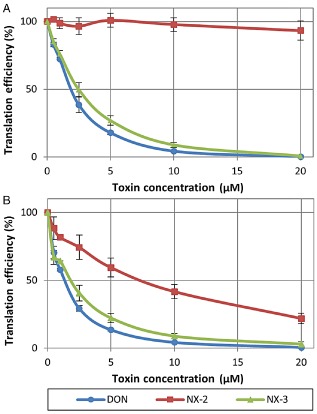
*In vitro* toxicity of deoxynivalenol and the novel metabolites NX‐2 and NX‐3 in (A) rabbit reticulocyte lysate and (B) wheat germ extract based translation assays. Error bars show ± standard deviations.

Trichothecenes are potent inhibitors of eukaryotic protein synthesis. However, derivatization at C‐3 alleviates toxicity of otherwise inhibitory trichothecenes. To prevent self‐intoxication of the producing fungi, the C3‐OH of trichothecenes is protected by *Tri101* mediated acetylation during biosynthesis and strongly reduced toxicity of 3‐ADON to mammalian ribosomes compared with DON has been documented (Kimura *et al*., 1998). The inhibitory activity of the novel substances on plant and mammalian ribosomes were tested using *in vitro* translation systems based on wheat germ extracts or rabbit reticulocyte lysates. NX‐3 showed translation inhibitor activity similar to DON in both assays (Fig. 3). NX‐2 did not inhibit ribosomes of the rabbit reticulocyte lysate, but seemed to be inhibitory for wheat ribosomes to a considerable extent. This might be due to different susceptibilities of plant and mammalian ribosomes towards NX‐2 or might be an artifact caused by de‐acetylation during the assay. Therefore, we determined the NX‐2 and NX‐3 concentrations in the translation assays. In wheat germ assays, 31% of NX‐2 was converted to NX‐3 during the incubation time, while only marginal deacetylation (< 1%) was observed with the rabbit reticulocyte lysate. From these observations, we conclude that NX‐2 is not inhibitory for both rabbit reticulocyte and wheat ribosomes, and the apparent toxicity for wheat ribosomes is fully explained by formation of NX‐3 by the wheat germ extract. Furthermore, the phytotoxicity of NX‐2 and NX‐3 using a *Chlamydomonas reinhardtii* growth assay was tested. Average generation times over 4 days using 100 μM of the trichothecene, were 21.5 h (NX‐2), 23.9 h (3‐ADON), 64.8 h (NX‐3) and over 96 h (DON) compared with 20 h for an acetone control (Fig. S3).

### Genetic basis of the NX chemotype

We intended to elucidate the cause for biosynthesis of NX‐2 instead of 3‐ADON. The *TRI1* genes of different *Fusarium* species encode rather distantly related cytochrome P450 monooxygenases (CYP), which are responsible for oxidation at C‐7 and/or C‐8 of the trichothecene scaffold (Alexander *et al*., 2009). While the Tri1 protein of *F. graminearum* was shown to hydroxylate both carbon atoms, the *F. sporotrichioides TRI1* gene product catalyses oxidation at C‐8 only. Thus, we hypothesized that the N‐isolates either contain a variant of *TRI1*, which is able to introduce the hydroxyl group at C‐7 only, or this gene is defective and another of the numerous CYP genes in the *Fusarium* genome has taken over this function. To test this, we first determined the sequences of 20 N‐isolates. A multilocus genotyping (MLGT) assay was used to confirm the identity of these isolates as *F. graminearum*. Furthermore, each of these isolates had single‐nucleotide polymorphisms within the main trichothecene biosynthetic gene cluster (*TRI3* and *TRI12*), predictive of the 3‐ADON trichothecene chemotype. The *TRI1* sequences of the N‐isolates were compared with those of *F. graminearum* PH‐1. Eighteen of the 20 N‐isolates shared identical *TRI1* sequences and the two remaining isolates (00‐556 and 06‐204) each differed from the major N‐isolate sequence type by only one unique nucleotide. There were no differences in the predicted amino acid sequences of the 20 N‐isolates. However, we identified 14 amino acid differences between the *TRI1* gene product of N‐strains and PH‐1 (Table 2).

**Table 2:** Amino acid differences between N‐isolates and PH‐1

Amino acid position	N‐isolate	PH‐1
33	T	A
100	N	S
115	L	F
210	T	S
252	S	R
254	M	L
256	N	T
346	I	F
361	F	I
363	I	V
373	E	D
418	K	Q
430	P	T
450	V	A

**Figure 4:**
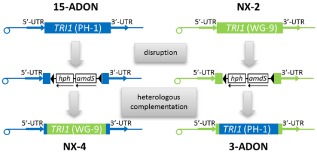
Schematic description of the *TRI* *1* allele swap between PH‐1 and 02‐264 (WG‐9).

To test whether the *TRI1* variant of the N‐isolates is indeed responsible for specific oxidation at C‐7, we constructed strains where the *TRI1* coding regions of PH‐1 and one N‐isolate are swapped (Fig. 4). In brief, we first deleted the *TRI1* genes in PH‐1 and isolate 02‐264 (lab designation WG‐9 for ‘wild grass’) by replacing a large part of their coding regions with the hygromycin resistance gene; the resulting strains were named IAPT (isogenic to PH‐1) and IAWT (isogenic to 02‐264, WG‐9). In the *tri1*Δ strains, neither DON, ADON nor NX‐2 could be detected, but they accumulated calonectrin, which is an intermediate of trichothecene biosynthesis and the substrate of Tri1p. This result is inconsistent with the hypothesis that another gene may be responsible for the C7‐hydroxylation. In the second step, we complemented the *TRI1* deletions with constructs carrying the other *TRI1* version. To this end, one *tri1*Δ strain of each genotype (IAPT10 and IAWT2) was transformed with a construct carrying a hybrid gene consisting of the core part of the *TRI1* from the opposite strain flanked by promoter and terminator regions of the receptor strain.

Five independent transformants were obtained for the PH‐1 background carrying the N‐version of *TRI1* (IAWP48, IAWP49, IAWP84, IAWP88, IAWP140). The heterologous complementation was confirmed by polymerase chain reaction (PCR) and restriction analysis (Fig. S4). The complementary experiment yielded one transformant (IAPW13), which, as expected, produced 3‐ADON and DON instead of NX‐2. This is consistent with the fact that the N‐strains have single‐nucleotide polymorphisms in the core TRI cluster that are predictive of the 3‐ADON chemotype. As expected for a 15‐ADON chemotype strain, PH‐1 produced 15‐ADON and DON, while in the WG‐9 control samples formation of NX‐2 was confirmed. One of the PH‐1 derived strains carrying the N‐*TRI1* allele (IAWP88) yielded a similar metabolite pattern as the parental *tri1*‐knockout strain IAPT10, indicating lack of expression for unknown reasons. Yet, in four out of the five PH‐1 derived strains containing the *TRI1* from WG‐9 (IAWP48, IAWP49, IAWP84 and IAWP140), two novel compounds were observed. One metabolite (named NX‐4, Fig. 1F) was predicted to be the 15‐acetyl equivalent of NX‐2 because PH‐1 is a 15‐ADON producer. While the MS/MS pattern of NX‐4 (Fig. S1C) was very similar to NX‐2, the retention times were different (9.5 min instead of 10.4 min). The other compound presumably is the 15‐acetyl‐equivalent of NX‐1. The candidate compound NX‐4 was purified from strain IAWP84 by reversed phase chromatography and was characterized by HRMS and NMR (see Table S1 for ^1^H‐NMR and in Table S2 for ^13^C‐NMR). NX‐4 was confirmed to be 15‐acetoxy‐3α,7α‐dihydroxy‐12,13‐epoxytrichothec‐9‐ene and the HRMS results were [M+H]^+^ calculated for C_17_H_24_O_6_, 325.1646; found, 325.1643; [M+NH_4_]^+^ calculated 342.1911; found, 342.1914; [M+Na]^+^ calculated 347.1465; found, 347.1466. Hence, with the *TRI1* swap between PH‐1 and the NX‐isolate, we verified that the N‐variant of Tri1p is responsible for the specific oxidation at position C‐7 alone.

## Discussion

We could demonstrate that the *F. graminearum* N‐strains, seemingly lacking toxin production and previously even considered as potential biocontrol strains (Yuen *et al*., 2010), in fact produce a new type A trichothecene. The new metabolite, named NX‐2, is formed in axenic cultures. It is similar to 3‐ADON but lacks the C‐8 keto group, which together with the conjugated C‐9,10 double bond creates the UV signal that is widely used by HPLC methods for detection of type B‐trichothecene contamination. Hence, NX‐2 escapes the analysis by HPLC‐UV based methods.

*In planta* NX‐2 is deacetylated to NX‐3, which is the equivalent of the well‐known mycotoxin DON without the C‐8 keto group. We demonstrated that in kernels of infected wheat much higher levels of the deacetylated toxin, NX‐3, compared with NX‐2, are found, similar to the situation with DON, where the acetylated derivatives are also typically present in levels less than 10% of that of DON.

Regarding toxicity, we could demonstrate that at the level of the ribosomal target, NX‐3 and DON have similar potency to inhibit protein synthesis. NX‐2 is not inhibitory in the tested concentration range, similar to the situation for its counterpart 3‐ADON. As the acetylated forms of DON are rapidly deacetylated after oral uptake in animals and humans, they are considered to be toxicologically equivalent to DON and were included in a group provisional maximum tolerable daily intake of 1 μg kg^−1^ body weight together with DON (World Health Organization, 2011). It is currently unknown to which extent the new compounds occur in naturally contaminated grain and whether they are therefore of potential toxicological relevance. It is also still unknown, whether or to which extent the new toxins are covered already due to cross‐reactivity by antibody‐based detection devices, which are widely used to screen for DON contamination of grain.

In a survey of strains conducted in the northern part of the US wheat‐growing areas highly affected by *Fusarium* (Minnesota, North and South Dakota) NX‐2 producing strains were isolated in only low frequency (approximately 3% of samples) (Liang *et al*., 2014). Some isolates, for instance the strain 02‐264, were isolated from non‐agricultural grasses. NX‐2 producers therefore may have a reservoir in plants other than wheat. At present, it is completely unclear how prevalent N‐strains are in other parts of the United States and whether they occur, for instance, also in Europe. The identified changes in the *TRI1* gene can be used to develop specific PCR‐based assays. Anecdotal evidence of grain with high levels of *Fusarium* damaged kernels, but low DON content might be explained by the presence of such strains, which so far escaped detection.

It will be important to monitor whether the frequency of N‐strains is changing, and most importantly to test whether they have a selective advantage on certain crop plants or genotypes. In the Northern United States and Canada, a shift in the chemotype composition of *F. graminearum* was observed and was directly tied to the spread of a novel, and potentially introduced, population of *F. graminearum* (Ward *et al*., 2008). Prior to 2000, strains from the United States and Canada were almost exclusively 15‐ADON producers; however, they have been increasingly replaced by 3‐ADON producers in some major wheat‐growing regions. The fact that all N‐strains characterized to date have core TRI clusters of the 3‐ADON type may indicate that the appearance of NX‐2 producing strains is connected to the recent expansion of the 3‐ADON population in North America.

NX‐2 and 3‐ADON producing strains primarily yield 3‐acetylated trichothecenes, which have reduced toxicity. It is possible that this allows for higher toxin production, as was observed among the 3‐ADON population in North America (Ward *et al*., 2008). We also observed a higher level of contamination with NX‐3 of the spray inoculated ‘Apogee’ compared with DON levels produced by the genetically unrelated PH‐1.

An intriguing hypothesis is that the fungi may benefit from protecting the C3‐OH of the toxin against plant detoxification enzymes, such as uridine diphosphate‐glucosyltransferases, residing in the plant cytosol. In the highly *Fusarium*‐affected spring wheat regions, wheat cultivars incorporating the Fhb1 resistance quantitative trait locus (QTL) are increasingly common (McMullen *et al*., 2012). It has been shown that the Fhb1 locus colocalizes with the ability to efficiently convert DON into DON‐3‐O‐glucoside (Lemmens *et al*., 2005), which is far less inhibitory to wheat ribosomes (Poppenberger *et al*., 2003).

The new toxin NX‐2 in addition to potentially escaping glycosylation may provide another advantage for the fungus. It has been shown that a glutathione adduct of DON can be formed (Gardiner *et al*., 2010), and that this reaction actually occurs *in planta* (Kluger *et al*., 2013). Presumably, the bulky glutathione residue prevents interaction with the ribosomal target (Fruhmann *et al*., 2014). At present, it is unknown whether glutathione‐mediated detoxification of the fungal virulence factor DON is relevant for Fusarium resistance and selected by breeders. For the formation of the Michael adduct, it is necessary that the thiol group of glutathione can react with the α,β‐unsaturated ketone. The lack of the keto group therefore may allow to circumvent this detoxification pathway, which could provide a selective advantage to the fungus. The construction of near isogenic strains only differing in the *TRI1* (and *TRI8*) alleles is an important step towards further work aiming to test these hypotheses on wheat cultivars differing in defined QTL regions.

In the *TRI1* swapping experiment, we have also generated a new toxin, NX‐4, corresponding to 15‐ADON lacking the keto group. This compound at present is not known to occur naturally. Yet, because the *TRI1* locus is not linked with the core *TRI* cluster, such segregants should arise during crossing of NX‐2 producers and 15‐ADON producers. More intensive screening may reveal that such strains occur in nature. Likewise, it seems possible that outcrossing with NIV producers, which have an intact *TRI13* gene encoding a C4‐hydroxylating enzyme in the core cluster and are prevalent in Asian countries, could lead to formation of the analogues of NIV and fusarenon X (4‐acetyl‐NIV) lacking the keto group.

In summary, we have discovered that a fungus that is a major threat to cereal production can make previously unknown toxins. The most relevant questions for future research are whether these compounds occur already in amounts relevant for public health and, in the worst case, whether NX‐2 producers possess an adaptive advantage that may permit them to overcome progress made by plant breeders in the last decades.

## Experimental procedures

### Fungal strains

The following *F. graminearum* strains were used in this study: PH‐1 (NRRL 31084, FGSC 9075), 00‐556 (NRRL 66047), 02‐264 (WG‐9, NRRL 66037), 06‐146 (NRRL 66030), 06‐156 (NRRL 66038), 06‐171 (NRRL 66036) and 06‐204 (NRRL 66033). Details regarding their origin and construction of derived strains can be found in Table S3.

### Trichodiene measurements by GC‐MS

To verify an active Tri5 protein, required for trichothecene synthesis, the presence of trichodiene, the volatile precursor of trichothecene mycotoxins, was monitored in the HS of cultures from N‐isolates of *F. graminearum* (02‐264, 06‐146, 06‐156 and 06‐171). Because of lack of an authentic standard, *F. graminearum* PH‐1 (Gaffoor *et al*., 2005) in which trichodiene was already previously detected and annotated (Jélen *et al*., 1995) was used as a reference. For each culture, 8000 spores were pipetted on 2 ml potato dextrose agar in HS vials and cultivated at 22°C in the dark for 60, 70, 90, 100, 120 and 140 h. They were inoculated at different time points to allow consecutive measurements of all fungal cultures in 1 day. The fungal cultures were flushed with synthetic air 6 hours prior to extraction. HS solid phase microextraction (SPME) GC‐MS measurements were performed using a Gerstel MPS2XL autosampler and Gerstel Maestro 1.3.20.41 (Mühlheim, Germany) for HS SPME and an Agilent GC 6890N coupled to 5975B inert XL MSD with Agilent MSD Chemstation G1701EA E.02.00.493 (all Agilent Technologies, Waldbronn, Germany) for GC‐MS. HS SPME was performed at 22°C for 52 min using a 50/30 μm divinylbenzene/carboxen/polydimethylsiloxane (DVB/CAR/PDMS), 23 gauge, 2 cm stable flex fibres (Gerstel). The analytes were separated on an Agilent DB5‐ms column (30 m × 0.25 mm, 0.25 μm) and introduced to the MS interface via a non‐coated guard column (0.5 m × 0.1 mm, Agilent Technologies). The abundances of the detected sesquiterpenes were determined using MetaboliteDetector (Hiller *et al*., 2009).

### Cultivation of *F* *. graminearum* on rice cultures and sample preparation for LC‐MS measurements

*Fusarium graminearum* PH‐1 (as control) and four N‐isolates (02‐264, 06‐146, 06‐156, and 06‐171) were routinely grown on Fusarium minimal medium (Leslie and Summerell, 2006). When appropriate, the following antibiotics were added: hygromycin B at 100 mg l^−1^ and G418 at 40 mg l^−1^. Strains were sporulated in mung bean broth (10 g mung beans per litre of water), and conidiospores were quantified by counting them in a Fuchs‐Rosenthal chamber. For mycotoxin production, strains were cultivated on solid rice media. Baby food jars (200 ml), containing 10 g rice from a local store and 10 ml reverse osmosis water, were incubated at room temperature for 1 h, autoclaved for 60 min at 121°C and inoculated with 10^5^ spores. Incubation was performed for 1, 2 or 3 weeks at 20°C, 55% humidity at constant illumination. Thereafter, the samples were homogenized using 40 ml of acetonitrile : water : acetic acid (79:20:1, v:v:v) by an Ultra‐Turrax T25 from IKA‐Werke (Staufen, Germany) and extracted for 90 min at room temperature on a GFL rotary shaker (Burgwedel, Germany). After separation by centrifugation (10 min, 3220 *g*), 1 ml of the supernatant was transferred to an HPLC vial for analysis.

### Screening for novel metabolite with LC‐MS

Screening was performed on an Agilent Technologies 1100 series HPLC system coupled to a QTrap LC‐MS/MS system from Applied Biosystems (Foster City, CA, USA). Using atmospheric pressure chemical ionization, a full scan from 200 to 800 Da (cycle time 1 s) using the third quadrupole in positive ionization was conducted. Two microlitres of the fungal extracts were injected. Chromatographic separation was achieved at 25°C with a flow rate of 0.75 ml min^−1^ on an Agilent Zorbax Eclipse XDB‐C8 column (150 × 4.6 mm, 5 μm). The eluents were composed of water : methanol (A: 20:80, v:v; B: 10:90, v:v), and both contained 5 mM ammonium acetate. After an initial holding time of 2 min at 0% B, a linear gradient to 100% B within the next 10 min was applied, followed by a holding period of 3 min at 100%, a quick switch to 0% B at 15 min and a column equilibration for 5 min at 0% B. The following MS settings were used: source temperature 450°C, curtain gas 35 psi, sheath gas (GS1) 60 psi, drying gas (GS2) 15 psi, interface heater on, corona discharge current 2 μA, declustering potential 20 V. Data were acquired and analysed using Analyst 1.5.2. software from AB Sciex.

### Purification of NX‐2

For purification of NX‐2 rice cultures of isolate 06‐156 were cultivated for 24 days as described above and stored at −20°C until further processing. Each rice culture was homogenized with 80 ml ethyl acetate using an Ultra‐Turrax and then extracted for 90 min at room temperature on a rotary shaker. The extracts were pooled and evaporated to dryness on silica gel using a rotary evaporator at room temperature to avoid formation of NX‐1. Normal phase chromatography was performed using a 40 × ca. 800 mm self‐packed silica gel (63–200 μm) column, and petroleum ether : ethyl acetate (3:1, v:v) was used for packing and washing. NX‐2 was eluted with petroleum ether : ethyl acetate (1:1, v:v), dried down at room temperature using a rotary evaporator and reconstituted in methanol : water (20:80, v:v). Preparative HPLC was performed on an Agilent Technologies 1100 series system using a Gemini NX C18 column (150 × 21.5 mm, 5 μm) and a guard column of the same material from Phenomenex (Aschaffenburg, Germany). A linear methanol‐water gradient comprising of an initial 1 min hold time of 44% methanol and reaching 100% within further 5 min was applied. NX‐2 was collected with a peak‐based threshold at around 5 min. Chemstation B04.03 was used for acquisition. The fractions containing NX‐2 were pooled and freeze dried after removal of methanol using a rotary evaporator at room temperature. Twenty‐five milligrams of a white powder (purity > 95% according to LC‐UV at 200 nm) were obtained from 42 rice cultures.

### HRMS/MS characterization

HRMS/MS measurements were performed on a 1290 UHPLC system coupled to a 6550 iFunnel QTOF LC‐MS instrument from Agilent Technologies. Chromatographic separation was performed on an Agilent Zorbax SB C18 column (150 × 2.1 mm, 1.8 μm) at 30°C and a flow rate of 250 μl min^−1^. The eluents were composed of water (A) and methanol (B) containing both 0.1% formic acid and 5 mM ammonium formate. After an initial holding time at 10% B for 0.5 min, the percentage of B was linearly increased to reach 100% B at 10 min. Thereafter, the column was flushed at 100% for 2 min before switching back to the starting conditions at 12.1 min, and column equilibration at 10% B was performed until 15 min. The MS parameters were as follows: gas temperature 130°C, drying gas flow 15 l min^−1^, nebulizer 30 psig, sheath gas temperature 300°C and flow 10 l min^−1^, capillary voltage 4000 V and nozzle voltage 500 V. MS full scan was performed from 50 to 1000 Da, and targeted product ion scans were performed from 50 to 400 Da, both at a scan rate of 3 spectra s^–1^. The precursor ions were isolated using an isolation width of 1.3 *m/z*. Prior to use, the QTOF was tuned and calibrated, and during the run two references masses (*m/z* 121.0508 and *m/z* 922.0098) were constantly infused via a second nebulizer to ensure mass accuracy. MassHunter Workstation LC/MS Data Acquisition version B.05.01 and MassHunter Workstation Qualitative Analysis version B.06.00 were used for data handling.

### NMR characterization of NX‐2, NX‐3 and NX‐4

NMR spectra were obtained from CD_3_OD solutions on an Avance DRX‐400 FT‐NMR spectrometer (Bruker BioSpin GmbH, Rheinstetten, Germany) at ambient temperature using a 5 mm inverse broadband z‐gradient probe head. Chemical shifts were established on the basis of residual solvent resonances (3.31 p.p.m. for ^1^H, 49.15 p.p.m. for ^13^C). All pulse programs were taken from the Bruker software library. The NMR data were evaluated using TopSpin 1.3 (Bruker BioSpin GmbH).

### Production and purification of NX‐3

Part of the purified NX‐2 was dissolved in methanol : water (50:50, v:v) and mixed with the equal volume of 1% aqueous Na_2_CO_3_. Every 20 min, the conversion was checked by LC‐MS measurements on the QTrap‐LC/MS system, and after 200 min the reaction was stopped by the addition of acetic acid. Preparative HPLC purification was performed immediately afterwards using a Gemini NX C18 column (150 × 21.5 mm, 5 μm, Phenomenex) and a guard column of the same material using a methanol‐water gradient. After an initial hold time of 1 min with 20% methanol, the methanol content was linearly increased to 60% methanol within the next 9 min, and NX‐3 was collected by time‐based fractioning between 5.95 and 6.25 min. The fractions containing NX‐3 were pooled. Methanol was evaporated by a rotary evaporator at room temperature, and the remainder was freeze dried. Approximately 2.0 mg NX‐3 (purity > 99% according to LC‐UV at 200 nm) were gained from 2.9 mg of NX‐2.

### Formation of new trichothecenes in planta

The highly sensitive wheat line ‘Apogee’ was spray inoculated with spore suspensions of *F. graminearum* PH‐1 and four different N‐isolates (02‐264, 06‐146, 06‐156 and 06‐171). After incubation at 25°C for 11, 16 and 22 days the wheat heads were removed from the stems and frozen at −80°C until further processing. The samples were ground using a Retsch ball mill (Haan, Germany) for 1 min and were subsequently extracted with the fourfold amount of acetonitrile : water : acetic acid (79:20:1, v:v:v) for 60 min at 20°C on a rotary shaker for LC‐MS/MS analysis. They were centrifuged for 10 min at 20°C and 4000 *g*; the supernatant was transferred to an Eppendorf tube and centrifuged again for 5 min at 10 000 *g* before being transferred to an HPLC vial. The samples were stored at −20°C until analysis.

### LC‐MS/MS measurements of inoculated wheat

With the obtained standards for NX‐2 and NX‐3, the compounds were optimized on a 4000 QTrap LC‐MS system from AB Sciex (Foster City, CA, USA) using positive and negative electrospray ionization. A selected reaction monitoring (SRM) method in negative ionization mode was developed and used for the analysis of the infected wheat ears. Chromatographic separation was achieved using an Agilent Technologies 1290 UHPLC system and a Gemini C18 column (150 × 4.6 mm, 5 μm, Phenomenex) at 25°C and a flow rate of 800 μl min^−1^. The eluents were composed of water : methanol (A: 20:80, v:v, B: 3:97, v:v) both containing 5 mM ammonium acetate. After an initial holding period of 0% B for 1 min, the gradient was linearly increased to 100% B within the next 10 min. The column was flushed for 2.5 min at 100%, followed by a fast switching back to the initial conditions and holding these for 2.5 min; hence, the total run time was 16 min. For the SRM transitions (summarized in Table S4), the applied dwell time was 25 ms. The following MS settings were used: source temperature 550°C, curtain gas 30 psi, sheath gas (GS1) 50 psi, drying gas (GS2) 50 psi, interface heater on. Data were acquired and analysed using analyst 1.5.2. software from AB Sciex.

### GC‐MS characterization of inoculated wheat

A wheat spikelet inoculated with strain 02‐264 was weighed, 2 ml volume of acetonitrile : water (84:16, v:v) were added and the vial was shaken on an Eberbach reciprocal shaker (Ann Arbor, MI, USA) at room temperature for 24 h. The extract was passed through a minicolumn packed with C18 and aluminum oxide (1:3, w:w). An aliquot (1.5 ml) of the filtrate were evaporated to dryness under nitrogen and 25 μl derivatization reagent (trimethylsilylimidazole: TMS chloride = 100:1, v:v) were added. After 10 min of shaking, 200 μl isooctane containing 4 mg l^−1^ of mirex (internal standard) were added and shaken gently, after which 200 μl of HPLC water was added. The vial was vortexed and the clear upper isooctane layer was transferred to a GC vial with a 200 μl glass insert. The GC‐MS analysis was performed on a Shimadzu GCMS‐QP2010 (Kyoto, Japan). Perfluorotributylamine was used to tune the mass spectrometer, and an Agilent DB‐5ms capillary column (30 m × 0.25 mm, 0.25 μm) was used in the GC system. A high‐pressure injection method (300.0 kPa, 1.00 min) was used in the splitless injection system. Linear velocity of flow control mode was used with the following oven temperature programme: 150°C for 1 min, increase with 30°C min^−1^ to 280°C and holding for 5 min. Injection, ion source and interface temperatures were kept at 260, 250 and 280°C respectively. Injection volume was 1 μl. The data were collected from *m/z* 50 to 550 in positive ion electron ionization (EI) mode with EI energy of 70 eV. High‐resolution accurate mass measurements were performed in positive ion EI and PCI modes using a Finnigan MAT 95 double‐focusing instrument (Thermo Fisher Scientific, Waltham, MA, USA) coupled with a HP5890 Series II gas chromatograph (Agilent Technologies) with the same capillary column. The instrument resolution was set at 4000 (10% valley). Perfluorokerosene was used as internal reference for EI. The reagent gas for PCI was 4% ammonia in methane.

### *In vitro* translation assays

Coupled *in vitro* transcription/translation systems [TnT^®^ T7 coupled Rabbit Reticulocyte Lysate System and TnT^®^ T7 Coupled Wheat Germ Extract System both from Promega (Madison, WI, USA)] were used for the assays. Standard transcription/translation reactions were performed in a total volume of 15 μl according to the manufacturer's instructions in the presence of the respective compounds in 0.2% methanol (final concentration). First, the ribosomes were pre‐incubated with buffer, amino acids, DNA and inhibitor for 7 min, and T7‐RNA polymerase was added to start the coupled *in vitro* transcription/translation. The reactions were stopped by addition of 1 μl of a 1 mM cycloheximide solution after 24 min in case of reticulocyte lysate or after 30 min for the wheat germ assays respectively. Efficiency of translation was determined by measuring activity of the firefly luciferase reporter using the Promega Steady‐Glo^®^ Luciferase Assay System and the 2300 EnSpire^®^Multimode Plate Reader from Perkin‐Elmer. At least three independent assays using individual dilutions were performed for each substance. To determine deacetylation of NX‐2 during the *in vitro* translation assay, assays were set up as described above in 25 μl reaction volumes. At defined three time points, 5 μl samples were removed from all reactions and added to 45 μl acetonitrile. In case of wheat germ extract system, these were 9, 22 and 36 min, for the reticulocyte lysate samples were drawn at 8, 15 and 30 min. After centrifugation (10 min at 4°C and 21 000 *g*), samples were prepared for LC‐MS/MS analysis.

### *Chlamydomonas reinhardtii* toxicity assays

*Chlamydomonas reinhardtii* wild‐type strain CC‐125 mating type + was obtained from the Chlamydomonas Genetics Center (Department of Botany, Duke University, Durham, NC, USA). The phytotoxicity of individual trichothecenes was tested as described previously (Alexander *et al*., 1999). Triplicate 10 ml liquid cultures were initiated with 1 × 10^5^ cells ml^−1^ on a high‐salt, high‐acetate medium (Harris, 1989) containing 100 μM concentration of an individual trichothecene and grown for 4 days at room temperature, with shaking at 200 r.p.m. Trichothecenes were dissolved in acetone and the final concentration of acetone was less than 1%. Generation times were calculated from the number of culture doublings (log of the final density – log of the initial cell density)/log (2) over 4 days.

### Genetic basis of the NX chemotype

*Fusarium graminearum* DNA was extracted as published (Umpierrez‐Failache *et al*., 2013). Species identity and the genotype profile of the main trichothecene biosynthetic gene cluster were determined simultaneously via MLGT as described previously (Ward *et al*., 2008). Amplification of the *TRI1* region was performed in 25 μl volumes with 1× High Fidelity PCR buffer (Invitrogen Life Technologies, Carlsbad, CA, USA), 2 mM MgSO_4_, 0.2 mM concentration of each deoxynucleoside triphosphate, 0.6 μM concentrations of primers Tri16‐IF1 and Fg_Tri1‐R1, 1.0 units of Platinum Taq DNA Polymerase High Fidelity (Invitrogen Life Technologies) and 200 ng of genomic DNA. PCR consisted of an initial denaturation of 120 s at 96°C, followed by 35 cycles of 30 s at 94°C, 30 s at 53°C and 180 s at 68°C. PCR products were purified using Montage PCR96 Cleanup filter plates from Millipore (Billerica, MA, USA). Sequencing reactions were carried out according to the method of Platt and colleagues (2007) using internal primers. Sequencing reaction products were purified using BigDye XTerminator (Applied Biosystems) and analysed with an Applied Biosystems ABI 3730 DNA Analyzer. DNA sequences were assembled and edited with Sequencher (version 4.10, Gene Codes, Ann Arbor, MI, USA), and were aligned with the *TRI1* locus from the whole genome shotgun sequence of PH‐1 (locus tag FG00071.1) using the MUSCLE algorithm implemented in mega (Tamura *et al*., 2013). Amino‐acid sequences were inferred based on the predicted *TRI1* coding DNA sequence of PH‐1.

### Disruption and heterologous complementation of *TRI1* in PH‐1 and 02‐264 (WG‐9)

*Fusarium graminearum* strains PH‐1 and 02‐264 (WG‐9) were routinely grown and sporulated as described above. Transformation of *F. graminearum* was essentially performed as described (Gaffoor *et al*., 2005) with the following minor modifications: 100 ml yeast extract peptone dextrose were inoculated with 2.5 to 5 × 10^6^ conidia from mung bean broth and incubated with shaking (180 r.p.m.) at 20°C; after separation from remaining mycelium protoplasts were pelleted and washed once with STC buffer (containing sorbitol, Tris‐HCl and CaCl_2_); 10^7^ protoplasts were transformed with a total of 10 μg DNA in a total volume of 6 ml, and 600 μl portions were transferred to 15‐ml aliquots of regeneration medium and poured into Petri dishes. After 2 h of regeneration, each culture was overlayed with 15 ml of regeneration medium containing twofold concentration of antibiotic (200 mg l^−1^ hygromycin B or 80 mg l^−1^ G418 respectively). After 4–8 days, resistant strains were transferred to Fusarium minimal medium plates containing the appropriate amount of antibiotic. Each transformant was sporulated and plated to single colonies on selective media for two times to ensure genetically pure clones. For PCR of genomic DNA, small amounts of freshly grown mycelium were re‐suspended in 50 μl Tris pH 7.5, heated at full power in a microwave oven for 45 s. Two microlitres were used for PCR.

Genomic DNA was prepared from both strains and the *TRI1* coding region (including introns) and 3′ flanking region was amplified using primers Xba‐FgTRI1‐fw and FgTRI1.Kpn‐rv and cloned into pAB86. The resulting plasmids were termed pAB206 and pRS21. Tables S5 and S6 provide detailed information about the oligonucleotides used.

For disruption of the *TRI1* genes in PH‐1 and WG‐9, the flanking regions of both *TRI1* alleles were first cloned into plasmid pASB42, derived from pUG6 (Güldener *et al*., 1996). Approximately 750 bp from the *TRI1* promoter regions of PH‐1 or WG‐9 were amplified with primer pair TRI1(PH‐1)Δup_SfiI‐fw and TRI1Δup_SpeI‐rv or TRI1(WG‐9)Δup_SfiI/BanI‐fw and TRI1Δup_SpeI‐rv, respectively, using genomic DNA from PH‐1 and WG‐9. The resulting fragments were digested with SfiI and SpeI and cloned into pASB42 cleaved with the same enzymes, yielding plasmids pGW1039 (PH‐1) and pGW1047 (WG‐9). Similarly, the 3′ regions of both *TRI1* alleles were cloned using primer pairs TRI1Δdown_SalI‐fw and TRI1(PH‐1)Δdown_HindIII‐rv or TRI1Δdown_SalI‐fw and TRI1(WG‐9)Δdown_HindIII‐rv, respectively, for amplification and the restriction enzymes SalI and HindIII for cloning. The resulting plasmids were named pGW1049 (PH‐1) and pGW1052 (WG‐9). The four plasmids were used as templates for production of fragments used for split marker disruption. The constructs used for transformation of PH‐1 and WG‐9 were produced by PCR employing primer pairs TRI1(PH‐1)‐560_fw and Hph_PstI_fw or TRI1(WG‐9)‐546_fw and Hph_PstI_fw, respectively, for the 5′ flanking regions with approximately two thirds of the C‐terminal part of the *hph* selection marker and primer pairs TRI1(PH‐1)+2136_rv/ TRI1(WG‐9)+2108_rv and Hph_SacII_rv for an overlapping piece of *hph* plus the 3′ flanking regions of both *TRI1* alleles. Approximately 10 μg of the PCR products in equimolar amounts were used in the transformation of PH‐1 and WG‐9. Transformants were selected on 100 mg l^−1^ hygromycin B. The gene replacement was confirmed by triplex PCR using the following primer combinations: TRI1(PH‐1)Δup_SfiI‐fw and TRI1+168_rv and Hyg3_fw (PH‐1 5′‐end), TRI1(PH‐1)+1484_fw and TRI1(PH‐1)+2250_rv and AmdS‐rv (PH‐1 3′‐end), TRI1(WG‐9)‐640_fw and TRI1+168_rv and Hyg3_fw (WG‐9 5′‐end), and TRI1(WG‐9)+1476_fw and TRI1(WG‐9)+2298_rv and AmdS‐rv (PH‐1 3′‐end). Transformants were purified by several cycles of sporulation and plating to single colonies.

For heterologous complementation plasmids containing the *TRI1* coding region from WG‐9 between the flanking regions of the PH‐1 *TRI1* and *vice versa* were constructed as follows: first, plasmids pGW1054 (hph flanked by 5′ and 3′ regions of the *TRI1* from PH‐1) and pGW1055 (hph between 5′ and 3′ flanking regions of *TRI1* from WG‐9) were cloned from plasmids pGW1039 and pGW1049 or pGW1047 and pGW1052 respectively. Plasmids pGW1054 and 1055 were cleaved with Pfl23II (SplI) and BamHI and resulting backbones were cloned to Pfl23II (SplI) and BamHI fragments from pAB206 and pRS21, replacing the *hph* selection marker with the coding regions of *TRI1* from both strains. The resulting plasmids, pGW1056 and pGW1058, were digested with BanI and AsuI to release fragments containing only *F. graminearum* hybrid genes without plasmid DNA. In parallel, plasmid pRS37 containing an internal XhoI fragment of *PKS12* and the *nptII* selection marker was linearized within the *PKS12* open reading frame using the restriction enzyme StuI. Next, the *tri1*Δ strain IAPT10 (PH‐1) was co‐transformed with approximately 2.5 μg of the linearized pRS37 and 8–10 μg of the BanI‐AsuI fragment released from pGW1054. Similarly, IAWT2 (WG‐9 *tri1*Δ) was transformed with linearized pRS37 and the BanI‐AsuI fragment containing the *TRI1* (PH‐1) coding region between the WG‐9 flanking regions (Fig. 4). Transformants were selected on 40 mg l^−1^ G418. Resistant strains were screened for loss of the hygromycin resistance marker. A total of 170 G418 resistant PH‐1 transformants yielded five hygS strains (named IAWP48, IAWP49, IAWP84, IAWP88 and IAWP140), while one hygS transformant (IAPW13) was found among 26 WG‐9 transformants. To confirm that the *hph* marker was indeed replaced by the hybrid genes, PCRs were performed with the same primer combinations as described above for *TRI1* deletion. Furthermore, the *TRI1* genes were amplified from all transformants and from the parental strains PH‐1 and WG‐9 using primers Xba‐FgTRI1‐fw and FgTRI1.Kpn‐rv, and the resulting fragments were digested with ApoI (Fig. S4). Constructs and genomic regions relevant for disruption of *TRI1* and heterologous complementation are summarized in Fig. S5.

### Purification of NX‐4

Three 21‐day‐old rice cultures of the strain IAWP84 with swapped *TRI1* (Table S3) were extracted with ethyl acetate as described above. The extracts were pooled and evaporated to dryness at room temperature. After dissolving in acetonitrile, the extract was defatted two times with the 2.5 times amount of hexane. The acetonitrile layer was then directly injected into the preparative HPLC system. Separation was achieved on a SunFire OBD C18 column (100 × 19 mm, 5 μm, Waters, Eschborn, Germany) and a C18 guard column using a methanol water gradient. After an initial hold time of 1 min with 25% methanol, the methanol content was linearly increased to 70% methanol within the next 4 min. From 5.1 to 8 min, the column was flushed with 100% methanol before the column was equilibrated using the starting conditions. NX‐4 was collected by time‐based fractioning around 4.9 min. The fractions containing NX‐4 were pooled and freeze dried after removal of methanol using a rotary evaporator at room temperature. From three rice cultures, 7.5 mg of a white powder (purity > 90% according to LC‐UV at 200 nm) were obtained.

## Supporting information


**Fig. S1.** High‐resolution mass spectrometric product ion spectra of the novel compounds (A) NX‐2, (B) NX‐3 and (C) NX‐4 at a collision energy of 15 eV.
**Fig. S2.** Electron impact GC‐MS mass spectrum of TMS‐NX‐3. The weak molecular ion of *m/z* 498 (M^+^) is enhanced 20‐fold in the insert.
**Fig. S3.** *Chlamydomonas reinhardtii* grown in the presence of 100 μM trichothecenes. Average culture doublings after 4 days were 4.0 (3‐acetyl‐deoxynivalenol – 3‐ADON), 0.2 (deoxynivalenol – DON) and 4.5 (NX‐2), 1.5 (NX‐3) for the novel compounds, compared with 4.8 for an acetone control.
**Fig. S4.** PCR – restriction fragment length polymorphism (PCR RFLP) for confirmation of swapping of *TRI1* alleles. Left side: schematic representation of *TRI1* locus with primers used for amplification and the ApoI restriction sites. Right side: Restriction analysis of PCR fragments amplified from wild‐type controls and transformants.
**Fig. S5.** Constructs and genomic regions relevant for disruption of *TRI1* and heterologous complementation. (A) Schematic representation of plasmids used for production of DNA used for disruption and for construction of hybrid genes for heterologous complementation. (B) genomic region of *TRI1* wild‐type and (C) genomic region of *tri1* deletion. *Fusarium graminearum* genomic DNA is represented in grey, plasmid sequences and selection markers are given in black, small black boxes represent primers for PCR amplification (Table S2). PCR products are shown in orange (*TRI1* flanking regions), blue (for gene disruption) and red (for confirmation of *tri1* deletion).
**Table S1.** ^1^H NMR data (δ, p.p.m.; multiplicity; J, Hz).
**Table S2.** ^13^C NMR data (δ, p.p.m.).
**Table S3.** *Fusarium graminearum* strains used in this work including the genetic background and the source.
**Table S4.** Optimized MS and MS/MS parameters.
**Table S5.** List of oligonucleotides used in this work including the sequence and purpose.
**Table S6.** List of plasmids used in this work including the relevant characteristics, backbone, purpose and source.
