# Supplementary material for: New tricks of an old enemy: isolates of F usarium graminearum produce a type A trichothecene mycotoxin
Source: Environ Microbiol. 2015 Jan 30;17(8):2588–600. doi: 10.1111/1462-2920.12718 (PMC4950012; doi:10.1111/1462-2920.12718)
Supplement: Supplementary file 1 — Fig. S1. High‐resolution mass spectrometric product ion spectra of the novel compounds (A) NX‐2, (B) NX‐3 and (C) NX‐4 at a collision energy of 15 eV. Fig. S2. Electron impact GC‐MS mass spectrum of TMS‐NX‐3. The weak molecular ion of m/z 498 (M+) is enhanced 20‐fold in the insert. Fig. S3. Chlamydomonas reinhardtii grown in the presence of 100 μM trichothecenes. Average culture doublings after 4 days were 4.0 (3‐acetyl‐deoxynivalenol – 3‐ADON), 0.2 (deoxynivalenol – DON) and 4.5 (NX‐2), 1.5 (NX‐3) for the novel compounds, compared with 4.8 for an acetone control. Fig. S4. PCR – restriction fragment length polymorphism (PCR RFLP) for confirmation of swapping of TRI1 alleles. Left side: schematic representation of TRI1 locus with primers used for amplification and the ApoI restriction sites. Right side: Restriction analysis of PCR fragments amplified from wild‐type controls and transformants. Fig. S5. Constructs and genomic regions relevant for disruption of TRI1 and heterologous complementation. (A) Schematic representation of plasmids used for production of DNA used for disruption and for construction of hybrid genes for heterologous complementation. (B) genomic region of TRI1 wild‐type and (C) genomic region of tri1 deletion. Fusarium graminearum genomic DNA is represented in grey, plasmid sequences and selection markers are given in black, small black boxes represent primers for PCR amplification (Table S2). PCR products are shown in orange (TRI1 flanking regions), blue (for gene disruption) and red (for confirmation of tri1 deletion). Table S1. 1H NMR data (δ, p.p.m.; multiplicity; J, Hz). Table S2. 13C NMR data (δ, p.p.m.). Table S3. Fusarium graminearum strains used in this work including the genetic background and the source. Table S4. Optimized MS and MS/MS parameters. Table S5. List of oligonucleotides used in this work including the sequence and purpose. Table S6. List of plasmids used in this work including the relevant characteristics, backbone, purpose [file EMI-17-2588-s001.docx]

Supporting Information for

New tricks of an old enemy: Isolates of *Fusarium graminearum* produce a type A trichothecene mycotoxin

Elisabeth Varga^1, #^, Gerlinde Wiesenberger^2, #^, Christian Hametner^3^, Todd J. Ward^4^, Yanhong Dong^5^, Denise Schöfbeck^1^, Susan McCormick^4^, Karen Broz^5^, Romana Stückler^2^, Rainer Schuhmacher^1^, Rudolf Krska^1^, H. Corby Kistler^5,6^, Franz Berthiller^1,^* and Gerhard Adam^2^

^1^Christian Doppler Laboratory for Mycotoxin Metabolism and Center for Analytical Chemistry, Department for Agrobiotechnology (IFA-Tulln), University of Natural Resources and Life Sciences, Vienna (BOKU), Konrad Lorenz Str. 20, 3430 Tulln, Austria

^2^Department of Applied Genetics and Cell Biology, University of Natural Resources and Life Sciences, Vienna (BOKU), Konrad Lorenz Str. 24, 3430 Tulln, Austria

^3^Institute of Applied Synthetic Chemistry, Vienna University of Technology, Getreidemarkt 9/163-OC, 1060 Vienna, Austria

^4^Bacterial Foodborne Pathogens & Mycology Research Unit, National Center for Agricultural Utilization Research, United States Department of Agriculture, 1815 N. University Street, Peoria, IL 61604, United States

^5^Department of Plant Pathology, University of Minnesota, 1991 Upper Buford Circle, St. Paul, MN 55108, United States

^6^Agriculture Research Service, United States Department of Agriculture, 1551 Lindig Avenue, St. Paul, MN 55108, United States

^#^ These authors contributed equally to this work.

* **Corresponding author:** Franz Berthiller, Christian Doppler Laboratory for Mycotoxin Metabolism and Center for Analytical Chemistry, Department for Agrobiotechnology (IFA-Tulln), University of Natural Resources and Life Sciences, Vienna (BOKU), Konrad Lorenz Str. 20, 3430 Tulln, Austria

phone: +43 2272/66280-413, fax: +43 2272/66280-403, e-mail: [franz.berthiller@boku.ac.at](mailto:franz.berthiller@boku.ac.at)

SUPPORTING INFORMATION FIGURES





Supporting Information Fig. 1. High resolution mass spectrometric product ion spectra of the novel compounds (a) NX-2, (b) NX-3 and (c) NX-4 at a collision energy of 15 eV.





**Supporting Information Fig. 2.** Electron impact GC-MS mass spectrum of trimethylsilyl-NX-3.

The weak molecular ion of *m/z* 498 (M^+^) is enhanced 20 fold in the insert.

**
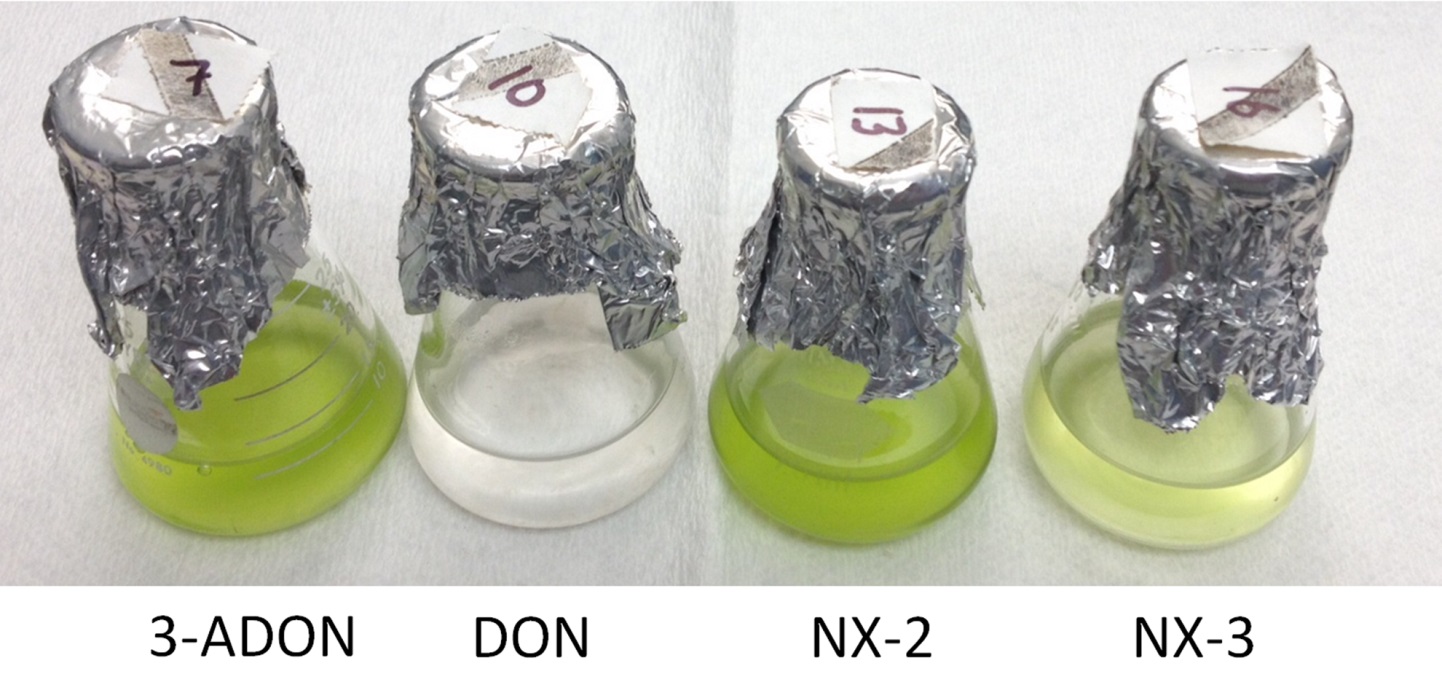
**

**Supporting Information Fig. 3.** *Chlamydomonas reinhardtii* grown in the presence of 100 µM trichothecenes. Average culture doublings after 4 days were 4.0 (3-acetyl-deoxynivalenol – 3-ADON), 0.2 (deoxynivalenol – DON), and 4.5 (NX-2), 1.5 (NX-3) for the novel compounds, compared to 4.8 for an acetone control.


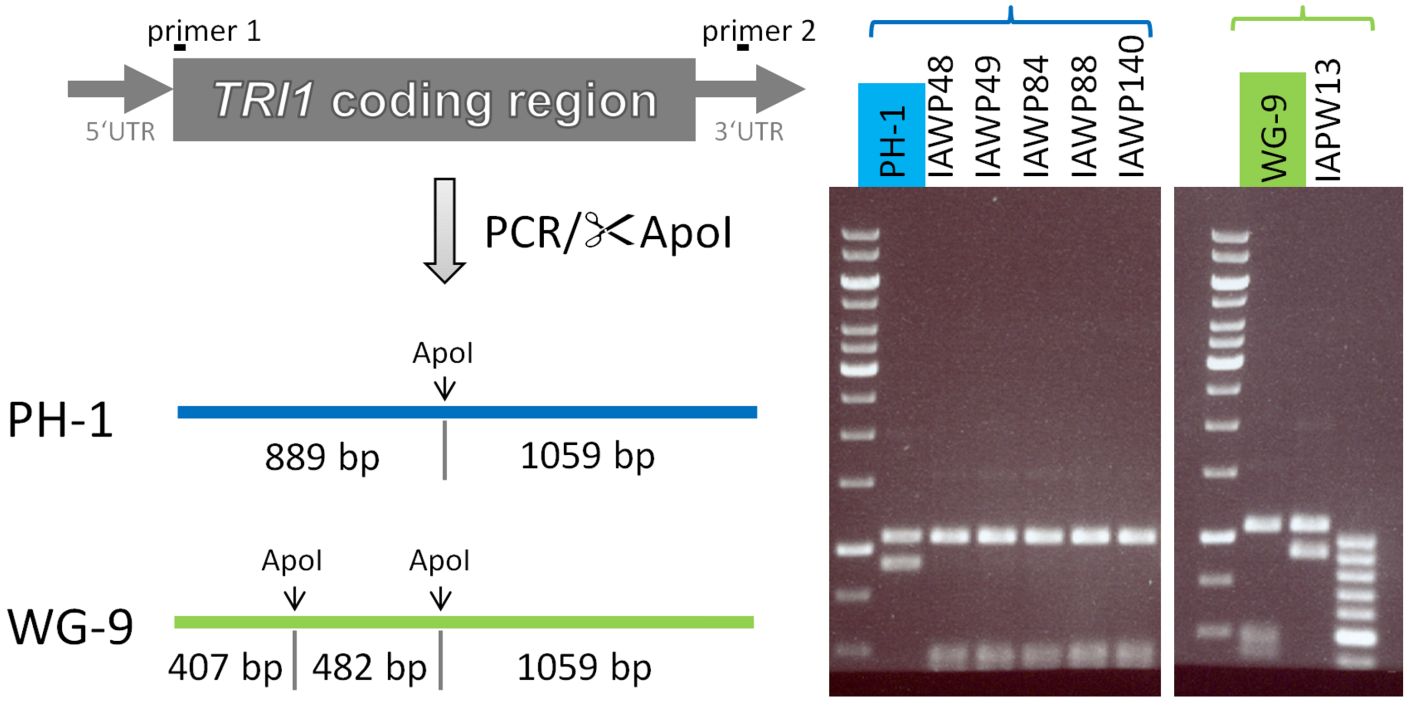


Supporting Information Fig. 4. Polymerase chain reaction – restriction fragment length polymorphism (PCR RFLP) for confirmation of swapping of *TRI1* alleles.

Left side: schematic representation of *TRI1* locus with primers used for amplification and the ApoI restriction sites. Right side: Restriction analysis of PCR fragments amplified from wild-type controls and transformants.


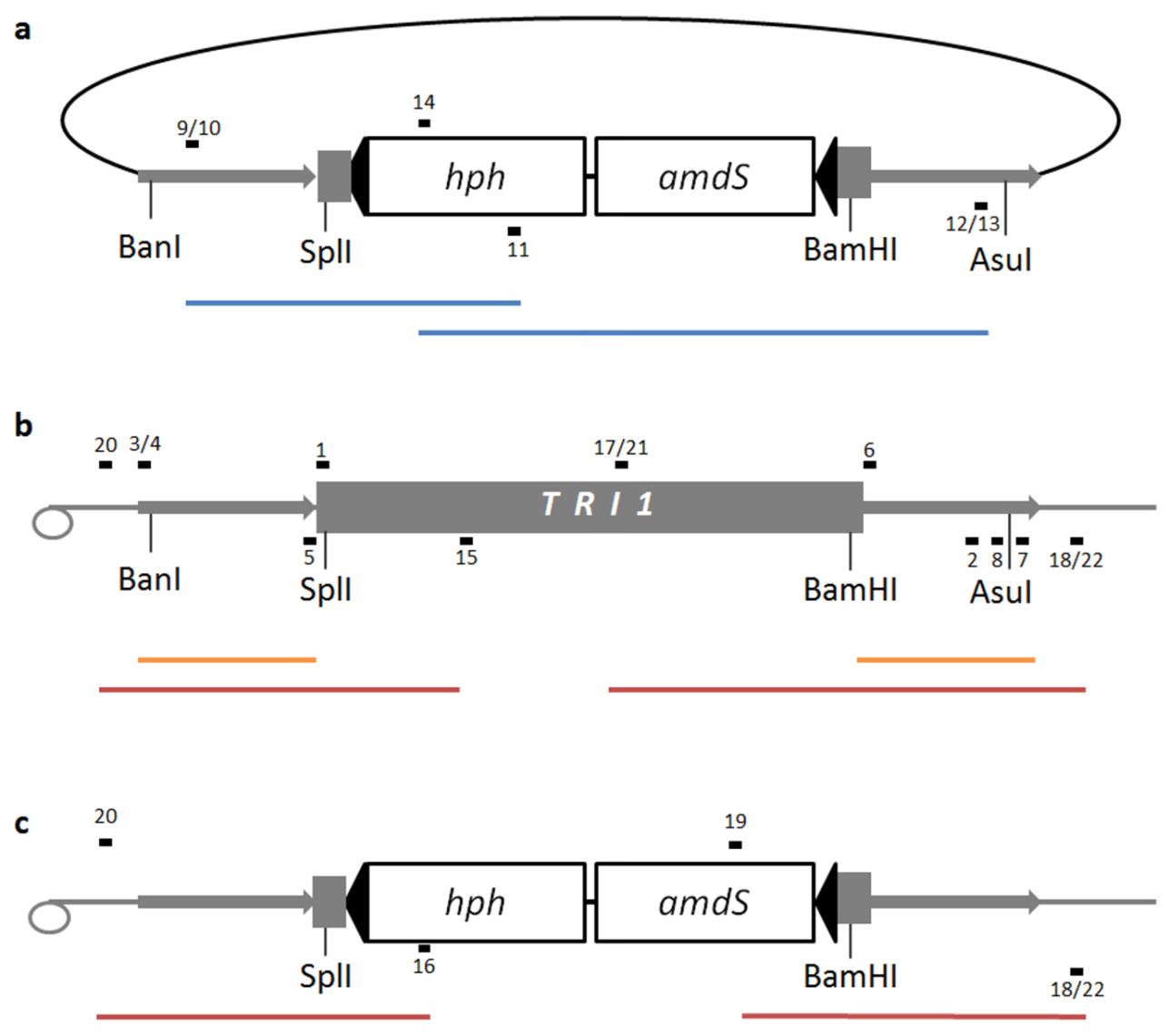


Supporting Information Fig. 5. Constructs and genomic regions relevant for disruption of *TRI1* and heterologous complementation.

(a) Schematic representation of plasmids used for production of DNA used for disruption and for construction of hybrid genes for heterologous complementation. (b) genomic region of *TRI1* wild-type and (c) genomic region of *tri1* deletion. *F. graminearum* genomic DNA is represented in grey, plasmid sequences and selection markers are given in black, small black boxes represent primers for PCR amplification (Table S2). PCR products are shown in orange (*TRI1* flanking regions), blue (for gene disruption) and red (for confirmation of *tri1* deletion).

SUPPORTING INFORMATION TABLES

Supporting Information Table 1. ^1^H NMR data (δ, ppm; multiplicity; J, Hz).

| Pos | NX-2 | NX-3 | NX-4 |
| --- | --- | --- | --- |
| 2 | 3.68 (d, 4.4) | 3.38 (d, 4.4) | 3.40 (d, 4.4) |
| 3 | 5.06 (dt, 11.4, 4.4) | 4.32 (dt, 11.1, 4.4) | 4.31 (dt, 11.1, 4.4) |
| 4 | 2.56 (dd, 14.9, 4.4)  2.05 (m) | 2.23 (dd, 14.5, 4.4)  1.98 (dd, 14.5, 11.1) | 2.49 (dd, 14.5, 4.3)  1.97 (dd, 14.5, 11.1) |
| 7 | 4.44 (dd, 9.0, 7.5) | 4.46 (dd, 9.1, 7.4) | 4.54 (dd, 10.3, 6.5) |
| 8 | 2.32-2.25 (m) | 2.35-2.30 (m) | 2.28 (dd, 17.8, 6.3)  2.12 (dd, 17.8, 10.4) |
| 10 | 5.36 (m) | 5.38 (m) | 5.45 (m) |
| 11 | 4.23 (d, 5.4) | 4.24 (d, 5.3) | 4.62 (d, 5.5) |
| 13 | 3.19 (d, 4.3)  3.08 (d, 4.3) | 3.16 (d, 4.4)  3.03 (d, 4.4) | 3.15 (d, 4.4)  3.03 (d, 4.4) |
| 14 | 1.26 (s) | 1.24 (s) | 1.11 (s) |
| 15 | 3.83 (d, 11.5)  3.76 (d, 11.5) | 3.78 (s) | 4.39 (d, 12.2)  4.20 (d, 12.2) |
| 16 | 1.74 (s) | 1.74 (s) | 1.73 (s) |
| 3-Ac | 2.08 (s) | - | - |
| 15-Ac | - | - | 2.03 (s) |

Supporting Information Table 2. ^13^C NMR data (δ, ppm).

| Pos | NX-2 | NX-3 | NX-4 |
| --- | --- | --- | --- |
| 2 | 80.3 | 82.3 | 82.3 |
| 3 | 73.1 | 69.9 | 69.7 |
| 4 | 42.9 | 46.0 | 46.1 |
| 5 | 47.2 | 47.9 | 47.4 |
| 6 | 48.2 | 48.3 | 47.8 |
| 7 | 71.0 | 71.6 | 69.3 |
| 8 | 40.6 | 40.7 | 40.4 |
| 9 | 140.4 | 140.1 | 140.1 |
| 10 | 120.2 | 120.5 | 120.9 |
| 11 | 72.9 | 72.8 | 72.4 |
| 12 | 66.2 | 66.7 | 66.6 |
| 13 | 48.8 | 48.6 | 48.7 |
| 14 | 16.7 | 17.2 | 15.9 |
| 15 | 62.6 | 63.1 | 63.8 |
| 16 | 22.9 | 22.9 | 22.9 |
| 3-Ac | 172.6  20.9 | - | - |
| 15-Ac | - | - | 172.7  21.1 |

**Supporting Information Table 3.** *Fusarium graminearum* strains used in this work including the genetic background and the source.

| **name** | **relevant genotype** | **parental strain** | **origin** | **source** |
| --- | --- | --- | --- | --- |
| PH-1 | 15-ADON | - | - | NRRL 31084 |
| 00-556^a^  02-264^a,b^  06-146^a^  06-156^a^  06-204^a^  06-171^a^ | 3-ADON  3-ADON  3-ADON  3-ADON  3-ADON  3-ADON | -  -  -  -  -  - | MN, Polk County  MN, Cook County  MN, Douglas County  MN, Douglas County  MN, Yellow Medicine County  MN, Douglas County | NRRL 66047  NRRL 66037  NRRL 66030  NRRL 66038  NRRL 66033  NRRL 66036 |
| IAPT10 | *tri1*Δ::*hph* | PH-1 | - | this work |
| IAWT2 | *tri1*Δ::*hph* | WG-9 | - | this work |
| IAWP48 | *TRI1*(WG-9) | PH-1 | - | this work |
| IAWP49 | *TRI1*(WG-9) | PH-1 | - | this work |
| IAWP84 | *TRI1*(WG-9) | PH-1 | - | this work |
| IAWP88 | *TRI1*(WG-9) | PH-1 | - | this work |
| IAWP140 | *TRI1*(WG-9) | PH-1 | - | this work |
| IAPW13 | *TRI1*(PH-1) | WG-9 | - | this work |

^a^ The first two digits indicate the year of isolation of the strain.

^b^ Also referred to as WG-9, isolated from wild grass.

**Supporting Information Table 4.** Optimized ESI-MS and ESI-MS/MS parameters.

| **compound** | **precursor ion (*m/z*) /  declustering potential (V)** | **product ions (*m/z*) /  collision energy (V)** |
| --- | --- | --- |
| deoxynivalenol | 355.1 [M+CH_3_COO]^-^ / -40 | 265.2 / -22  59.2 / -40 |
| 3-acetyl-deoxynivalenol | 397.3 [M+ CH_3_COO]^-^ / -38 | 307.2 / -20  59.2 / -38 |
| NX-2 | 383.1 [M+ CH_3_COO]^-^ / -45 | 323.1 / -14  233.1 / -26  59.0 / -36 |
|  | 323.1 [M-H]^-^ / -80 | 233.0 / -18  73.0 / -34  59.0 / -30 |
| NX-3 | 341.1 [M+ CH_3_COO]^-^ / -50 | 137.0 / -20  281.0 / -14  59.0 / -36 |
|  | 281.1 [M-H]^-^ / -80 | 233.0 / -14  135.0 / -30 |
|  |  | 137.0 / -16 |

**Supporting Information Table 5.** List of oligonucleotides used in this work including the sequence and purpose.

| **name** | **#^a^** | **sequence** | **purpose** |
| --- | --- | --- | --- |
| Tri16-IF1 | **-** | 5’-GCCTSATAGCGACGATCTTGC-3’ | Amplification and sequencing of *TRI1* |
| Fg_Tri1-R1 | **-** | 5’-AACAAGTGGCGAGATCAAACC-3’ | Amplification and sequencing of *TRI1* |
| Xba-FgTRI1-fw | **1** | 5’-CTCTAGAATGGCTCTCATCACCAGTTTG-3’ | Amplification and cloning of *TRI1* from PH-1 and WG-9 |
| FgTRI1.Kpn-rv | **2** | 5’-ATGGTACCGTCAACAACTGGCGAGAT-3’ | Amplification and cloning of *TRI1* from PH-1 and WG-9 |
| TRI1(PH-1)Δup_SfiI-fw | **3** | 5’-TAATGGCCGCATAGGCCGTAGTTTCAAGGAGT GGTCG-3’ | Cloning of 5’ flanking region of *TRI1* from PH-1; Confirmation of disruption in PH-1 (5’ end) |
| TRI1(WG-9)Δup_SfiI/ BanI-fw | **4** | 5’-TAATGGCCGCATAGGCCGGTGCCGTTAGATGG CGGAAGAG-3’ | Cloning of 5’ flanking region of *TRI1* from WG-9 |
| TRI1Δup_SpeI-rv | **5** | 5’-AAACTAGTCTGGGGTTGAGCAAGTATG-3’ | Cloning of 5’ flanking region of *TRI1* from PH-1 and WG-9 |
| TRI1Δdown_SalI-fw | **6** | 5’-AATGTCGACGTGGAAGATTCCTGAAGGTC-3’ | Cloning of 3’ flanking region of *TRI1* from PH-1 and WG-9 |
| TRI1(PH-1)Δdown_HindIII-rv | **7** | 5’-ACAAGCTTAGTTTTCTTTCACTGTTGCATC-3’ | Cloning of 3’ flanking region of *TRI1* from PH-1 |
| TRI1(WG-9)Δdown_HindIII-rv | **8** | 5’-ACAAGCTTCGAACTAGGGATGATCCAACG-3’ | Cloning of 3’ flanking region of *TRI1* from WG-9 (AsuII site created) |
| TRI1(PH-1)-560_fw | **9** | 5’-CTATGAAGGTTACGTGTACTGG-3’ | Amplification of disruption construct for PH-1  (5’ flanking region+part of *hph*) |
| TRI1(WG-9)-546_fw | **10** | 5’-TCATAATCTCAAGGTCAACCGC-3’ | Amplification of disruption construct for WG-9  (5’ flanking region+part of *hph*) |
| Hph_PstI_fw | **11** | 5’-CTGTTCTGCAGCCGGTCGC-3’ | Amplification of disruption constructs for PH-1 and WG-9 (5’ flanking region+part of *hph*) |
| TRI1(PH-1)+2136_rv | **12** | 5’-TGTTGCATCAACTGGGTAATTCG-3’ | Amplification of disruption construct for PH-1  (3’ flanking region+part of *hph*) |
| TRI1(WG-9)+2108_rv | **13** | 5’-AGGGATGATCCAACGTGCTGC-3’ | Amplification of disruption constructs for PH-1 and WG-9 (3’ flanking region+part of *hph*) |
| Hph_SacII_rv | **14** | 5’-AGCCGCGGCGATCCTGCAAG-3’ | Amplification of disruption constructs for PH-1 and WG-9 (3’ flanking region+part of *hph*) |
| TRI1+168_rv | **15** | 5’-TGAGACTGCACGAGCATCG-3’ | Confirmation of disruption in PH-1 and WG-9  (5’ end) |
| Hyg3_fw | **16** | 5’-AGAAGTACTCGCCGATAGTG-3’ | Confirmation of *TRI1* disruption in PH-1 and WG-9  (5’ end) |
| TRI1(PH-1)+1484_fw | **17** | 5’-ACAAGGTCCAGCTATCTTCG-3’ | Confirmation of *TRI1* disruption in PH-1 (3’ end) |
| TRI1(PH-1)+2250_rv | **18** | 5’-TCAGATTTGAGGCTCATAAGG-3’ | Confirmation of *TRI1* disruption in PH-1 (3’ end) |
| AmdS-rv | **19** | 5’-ACACCTGCCGTGTCAGCC-3’ | Confirmation of *TRI1* disruption in PH-1 and WG-9  (3’ end) |
| TRI1(WG-9)-640_fw | **20** | 5’-CGAAACTATGAACAGGCTCC-3’ | Confirmation of *TRI1* disruption in WG-9 (5’ end) |
| TRI1(WG-9)+1476_fw | **21** | 5’-CAGAGAAGACAAGGTTCAGC-3’ | Confirmation of *TRI1* disruption in WG-9 (3’ end) |
| TRI1(WG-9)+2298_rv | **22** | 5’-GCCACATTCGCTAGTCTGC-3’ | Confirmation of *TRI1* disruption in WG-9 (3’ end) |

^a^ Numbers refer to primers indicated in Supporting Information Figures 2 and 4.

**Supporting Information Table 6.** List of plasmids used in this work including the relevant characteristics, backbone, purpose and source.

| **name** | **relevant characteristics** | **backbone** | **purpose** | **source** |
| --- | --- | --- | --- | --- |
| pAB86 | *pki* promoter, *gpdA* promoter-*hph*-*cbh2* terminator | pUC19 | expression vector | this work |
| pAB206 | *pki* promoter-*TRI1*(PH-1) | pAB86 |  | this work |
| pRS21 | *pki* promoter-*TRI1* (WG-9) | pAB86 |  | this work |
| pRS37 | *PKS12* (XhoI fragment), nptII | pII99 | co-transformation, selection on G418 | this work |
| pUG6 | *loxP-kanMX-loxP* |  |  | Güldener  et al.^1^ |
| pASB42 | *loxP-hph-amdS-loxP* | pUG6 | construction of plasmids for disruption | this work |
| pGW1039 | *TRI1* promoter (PH-1), *hph* | pASB42 |  | this work |
| pGW1047 | *TRI1* promoter (WG-9), *hph* | pASB42 |  | this work |
| pGW1049 | *TRI1* terminator(PH-1), *hph* | pASB42 |  | this work |
| pGW1052 | *TRI1* terminator (WG-9), *hph* | pASB42 |  | this work |
| pGW1054 | *TRI1* promoter and terminator (PH-1), *hph* | pASB42 |  | this work |
| pGW1055 | *TRI1* promoter and terminator (PH-1), *hph* | pASB42 |  | this work |
| pGW1056 | *TRI1* promoter and terminator (PH-1), *TRI1* coding region (WG-9) | pASB42 | heterologous complementation of *tri1*Δ in PH-1 | this work |
| pGW1058 | *TRI1* promoter and terminator (WG-9), *TRI1* coding region (PH-1) | pASB42 | heterologous complementation of *tri1*Δ in WG-9 | this work |

^1^ Güldener, U., Heck, S., Fiedler, T., Beinhauer, J. and Hegemann, J. H. (1996) A new efficient gene disruption cassette for repeated use in budding yeast. Nucleic Acids Res 24: 2519-2524.
